# Properties of Waterborne Polyurethane Conductive Coating with Low MWCNTs Content by Electrostatic Spraying

**DOI:** 10.3390/polym10121406

**Published:** 2018-12-19

**Authors:** Fangfang Wang, Lajun Feng, Guangzhao Li

**Affiliations:** 1 School of Materials Science and Engineering, Xi’an University of Technology, Xi’an 710048, China; wff1170111008@163.com (F.W.); lgz0414@163.com (G.L.); 2 Key Laboratory of Corrosion and Protection of Shaanxi Province, Xi’an 710048, China

**Keywords:** waterborne polyurethane, multi-walled carbon nanotubes, conductive coating, electrical conductivity, corrosion resistance, adhesive strength

## Abstract

Because flammable organic solvents are emitted during the construction process, oil-based conductive coatings generally result in potential safety problems. A high content of conductive mediums can also weaken the adhesive and protective abilities of existing conductive coatings. Therefore, an anticorrosive and conductive coating was prepared on Q235 steel substrate by spraying the multi-walled carbon nanotubes (MWCNTs)/waterborne polyurethane (WPU) dispersion with a low MWCNT content in this work. The effect of the MWCNT content on the electrical conductivity, corrosion resistance, and adhesive strength of the WPU conductive coating was investigated. It was concluded that a spatial network structure of MWCNTs-WPU was formed to make the coating structure more compact. The electrical conductivity, corrosion resistance, and adhesive strength of the WPU conductive coating first increased and then decreased as the MWCNT content increased. When the MWCNT content was only 0.2 wt % (which was far lower than that of the existing conductive coatings at 1 wt %), the coating began to conduct electricity; its resistivity was 12,675.0 Ω·m. The best combination property was the 0.3 wt % MWCNTs/WPU conductive coating. Its adhesive strength was 19.99% higher than that of pure WPU coating. Its corrosion rate was about one order of magnitude lower than that of pure WPU coating after being immersed in 3.5 wt % NaCl solution for 17 days.

## 1. Introduction

Most of the raw materials and products from petrochemical enterprises are corrosive and flammable, and sparks arising from triboelectric charging can cause flammable raw materials to catch fire easily. Conductive coatings can avoid the formation of electrostatic sparks by discharging static electricity. Therefore, anticorrosive and conductive coatings have been widely used in chemical- and oil-refining industries [1,2,3]. However, most of the existing conductive coatings are oil-based coatings, which catch fire easily because they emit flammable organic solvents during the construction process and, thus, cause potential safety problems. Additionally, the content of conductive mediums in existing conductive coatings is high, generally about 1 wt % [4,5,6,7]. The higher content of conductive mediums in the conductive coating generates interfaces between conductive mediums and polymer matrix after curing. Corrosive mediums can conveniently penetrate through these interfaces to the equipment surfaces and can deteriorate the protective ability of the conductive coating. In addition, nanoscale conductive mediums are prone to agglomeration [8,9], generally resulting in poorer adhesive strength between the conductive coating and the steel substrate and in its worst properties of corrosion resistance and electrical conductivity [10,11]. In order to solve the practical issues above, waterborne polyurethane (WPU) with a safe and reliable ability was used as a polymer matrix [12], and multi-walled carbon nanotubes (MWCNTs) with good properties of corrosion resistance and electroconductibility was used as a conductive medium [13,14]. A low content of MWCNTs was dispersed in WPU by magnetic stirring and ultrasonic dispersion to prepare the MWCNTs/WPU dispersion, which was sprayed on a Q235 steel substrate by an electrostatic spraying equipment to form a layer of anticorrosive and conductive coating. The technology of electrostatic spraying with good atomization capability did well preventing MWCNTs from agglomerating [15]. The effect of the MWCNT content on the electrical conductivity, corrosion resistance, and adhesive strength of the WPU conductive coating prepared by electrostatic spraying was investigated, and the results may provide a theoretical basis for preparing water-based conductive coating with low conductive mediums content by electrostatic spraying.

## 2. Materials and Methods

### 2.1. Experimental Materials

WPU was supplied by Jining Huakai Resin Co. Ltd., Jining, China. Its solid content and volatile organic compound (V.O.C) concentration were 35% and 253 g/L, respectively. The MWCNTs (FloTube 9000 series) were supplied by Beijing Tiannai Technology Co., Ltd., Beijing, China. The average diameter, average length, and tap density of the MWCNTs were 10–15 nm, 10 μm, and 0.03–0.15 g/cm^3^, respectively.

### 2.2. Preparation of Coatings

Q235 steel (50 × 20 × 2 mm) was used as the steel substrate and was roughened by a YX-6050A sand blasting device, Anbangruiyuxin Machine Technology Development Co. Ltd., Wuhan, China. The air pressure was controlled at 0.7 ± 0.1 MPa. The distance between the spray gun and the steel substrate was kept at 130 ± 20 mm. The time of sand blasting treatment was 35 ± 5 s.

In order to obtain uniformly dispersed MWCNTs/WPU dispersions, different contents of MWCNTs (0, 0.2, 0.3, 0.4, 0.5 and 0.6 wt %) were each dispersed in WPU liquid by an 85–2 magnetic stirring device (Hangzhou Instrument Motor Co., Ltd., Hangzhou, China) at a speed of 250 ± 50 r/min for 30 min, and then, the mixtures were each treated by a KQ-50B ultrasonic dispersion device (Kunshan Ultrasonic Instrument Co., Ltd., Kunshan, China) for 30 min.

The MWCNTs/WPU dispersions with different MWCNTs contents prepared above were each sprayed on Q235 steel substrates to form conductive coatings by a NEW KCI-CU801 electrostatic spraying equipment, Shenzhen Honghaida Instrument Co., Ltd., Shenzhen, China. The voltage of electrostatic spraying was set at 55 ± 5 KV. The pressure of the compressed air was kept at 0.65 ± 0.05 MPa. The distance between the spray gun and the Q235 steel substrate was controlled at 100 ± 20 mm. Finally, the samples (the Q235 steel with a layer of coating) were cured first at room temperature for 1 day and then at 70 °C for 24 h in an oven (Zhejiang Yuyao Yuandong CNC Instrument Factory, Yuyao, China). Thus, a layer of MWCNTs/WPU coating was completely coated on the Q235 steel substrate.

### 2.3. Measurements

#### 2.3.1. Electrical Conductivity

The electrical conductivity of the coating was evaluated by its resistivity. Its square resistance was measured at room temperature by a DY2101 Digital Multimeter (Duoyi Multimeter, Xian, China). Its thickness was tested by an HCC-18 magnetoresistive thickness meter (Shanghai Huayang Testing Instrument Co., Ltd., Shanghai, China). The average value of each variable was calculated by 6 data points. The resistivity of the coating was calculated according to Equation (1):

ρ = *R* × *d* (1)

where ρ is the resistivity (Ω·m), *R* is the square resistance (MΩ), and *d* is the thickness (μm).

#### 2.3.2. Corrosion Resistance

The polarization curve and electrochemical impedance spectroscopy (EIS) of the coating were studied at room temperature by a Ver4.2corr Test system (Wuhan Corr Test Co., Ltd., Wuhan, China) with a three-electrode cell. The sample was used as the working electrode, the Pt electrode was used as the auxiliary electrode, and the saturated calomel electrode was used as the reference electrode. The test area of the sample was 0.785 cm^2^. The samples were immersed in 3.5 wt % NaCl solution for 17 days before testing. It was time to test the polarization curve and EIS of the coating when the open circuit potential of the system was stable.

#### 2.3.3. Adhesive Strength

In according with ISO 4624:1978, the peel test was performed on the coating using a D2-5DL universal mechanical testing machine (Changchun Mechanical institute, Changchun, China) at room temperature. The coating was totally peeled off from the Q235 steel substrate during the test, and its adhesive strength to the Q235 steel substrate was calculated by Equation (2):

σ = *F*/*A* (2)

where σ is the adhesive strength (MP), *F* is the maximum load (N), *A* is the coating area (mm^2^).

#### 2.3.4. Fourier Transform Infrared Spectroscopy

The mixture of the coating and the KBr with a mass ratio of 1:100 was evenly grinded and then pressed for 2 min at 80 MPa to form a thin slice. An Alpha FTIR spectrometer (Bruker Optics, Germany) was used to characterize the molecular structure of the coating at room temperature. The scanning range was 4000–500 cm^−1^.

#### 2.3.5. Scanning Electron Microscope

The surface morphology of the coating was studied by a Merlin Compact SEM (Zeiss, Germany) to characterize the dispersion of the MWCNTs.

## 3. Results and Discussion

### 3.1. FTIR

**Figure 1:**
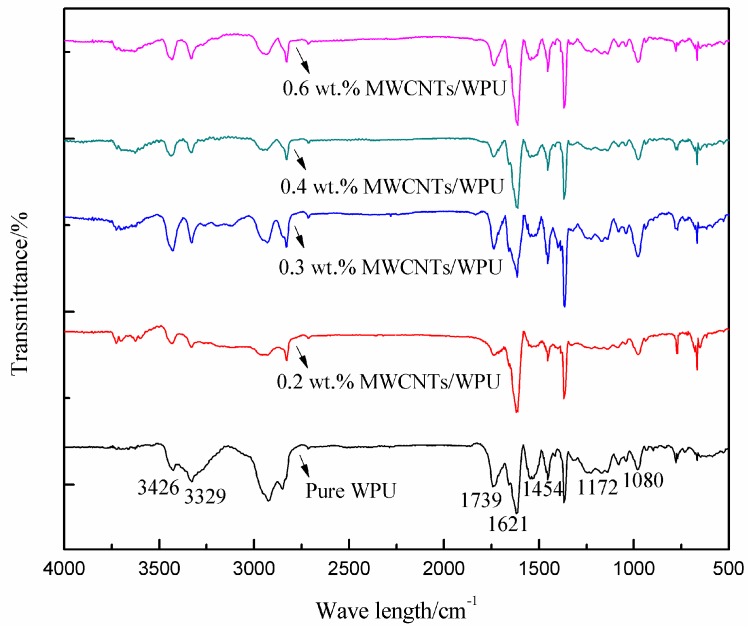
FTIR spectra of MWCNTs/WPU coatings with different MWCNTs contents.

The molecular structures of the WPU coatings with different MWCNT contents were characterized by FTIR Spectra (Figure 1). No significant stretching vibration absorption peak of the –NCO group at 2270 cm^−1^ was shown, indicating that it was completely involved in the polymerization reaction of the WPU during curing. In addition, there was no obvious change in the characteristic peak of the C–O bond at 1080 cm^−1^. The addition of MWCNTs in the WPU resulted in the significant weakening of the absorption peak of the N–H bond of different MWCNTs/WPU coatings at 3329 cm^−1^. However, the absorption peak of the C=O bond at 1621 cm^−1^ remarkably increased [16]. Under the action of high-voltage electrostatic, field the surface activity of MWCNTs was probably improved and then polar groups, such as –OH and –COOH groups, were absorbed on the surfaces of the MWCNTs [15,17]. The polymerization reaction that occurred between these polar groups with –NHCOO and –NCO groups in the molecular structure of WPU during curing consumed the N–H bond and formed a new C=O bond. Therefore, a spatial network structure of MWCNTs-WPU connected by chemical bonds was formed and made the structure of the conductive coating denser [18].

### 3.2. SEM

**Figure 2:**
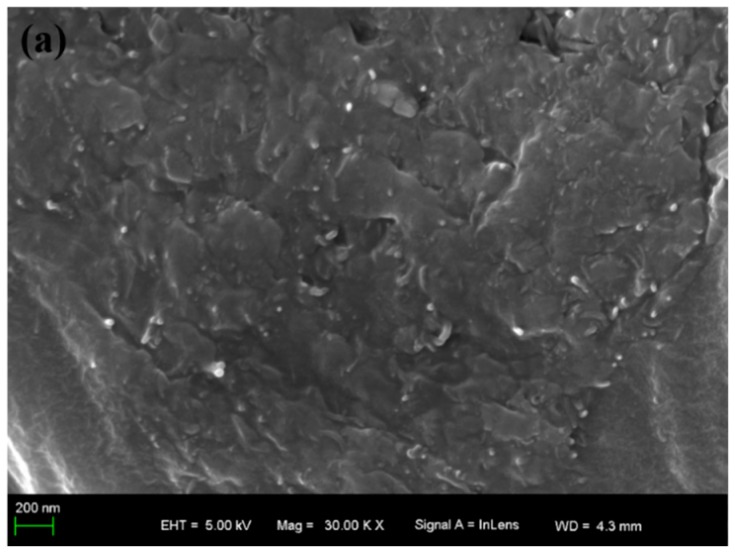

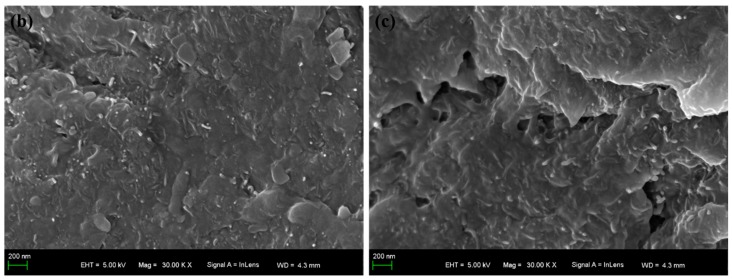
SEM images of (**a**) 0.2 wt % MWCNTs/WPU conductive coating, (**b**) 0.3 wt % MWCNTs/WPU conductive coating, and (**c**) 0.6 wt % MWCNTs/WPU conductive coating.

The surface morphology of the 0.2 wt % MWCNTs/WPU conductive coating (Figure 2a) shows that thanks to the technology of electrostatic spraying, the MWCNTs were evenly dispersed in the WPU matrix and the average gap between MWCNTs was within about a few nanometers. Therefore, MWCNTs could possibly form a valid conductive path even if the content was low. Comparing the surface morphology of 0.3 wt % (Figure 2b) with that of 0.6 wt % (Figure 2c) MWCNTs/WPU conductive coating, it was concluded that MWCNTs in both coatings contacted each other to generate a continuous conductive network [19]. The remarkable difference was that the structure of the former coating was more compact than that of the latter, probably because the addition of low MWCNT content should enhance the dispersibility of MWCNTs in WPU matrixes and the combination of MWCNTs/WPU interfaces and should reduce the micro-defects of the coating. However, an increase in the agglomerated MWCNTs by adding high MWCNT content may reduce the contact areas of MWCNTs/WPU interfaces, increase the micro-defects of the coating, and generate obvious macro-defects in the coating structure. The diversity in the surface morphologies of different MWCNTs/WPU conductive coatings will lead to a change in its properties [20,21].

### 3.3. Electrical Conductivity

Table 1 summarizes the resistivity of the MWCNTs/WPU conductive coating. As the MWCNT content increased from 0.2 to 0.6 wt %, the resistivity of the coating significantly decreased from 12,675.0 to 163.8 Ω·m, a decrease of about two orders of magnitude. The resistivity of the MWCNTs/WPU conductive coating listed in Table 1 was far less than the range of 10^8^–10^11^ Ω·m (the standard requirement of the conductive coating).

When the MWCNT content was only 0.2 wt %, the coating began to conduct electricity. However, the amount of MWCNTs was too small to contact with each other to become an electrical conductor probably because the average gap between MWCNTs is approximately within several nanometers so MWCNTs can form a л electron transport chain under the action of the voltage difference. As the MWCNT content increased from 0.3 to 0.5 wt %, the resistivity of the coating decreased by 96.9%, indicating a greatly enhanced electrical conductivity. The addition of MWCNTs at a certain critical value MWCNTs are highly likely to completely contact each other to form a continuous conductive network. The resistivity of the coating only decreased by 45.1% as the MWCNT content increased from 0.5 to 0.6 wt %. The reason for a more stable change in the resistivity of 0.5–0.6 wt % MWCNTs/WPU conductive coating compared to that of 0.3–0.5 wt % MWCNTs/WPU conductive coating may be that with MWCNT content above the critical value, the effect of the increasing MWCNT content on the electrical conductivity of the coating would be weaker.

### 3.4. Corrosion Resistance

#### 3.4.1. Polarization Curve Analysis

**Figure 3:**
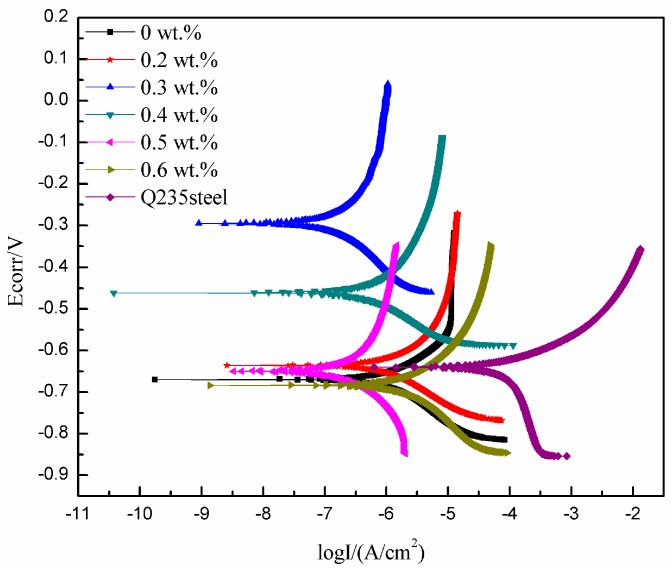
Polarization curves of different MWCNTs/WPU conductive coatings.

Figure 3 shows polarization curves of different MWCNTs/WPU conductive coatings after immersion in 3.5 wt % NaCl solution for 17 days. Table 2 lists the corrosion current density and corrosion rate of the corresponding coating fitted by computer software. Figure 3 and Table 2 shows that as the MWCNT content increased, the corrosion current density and corrosion rate of the coating first decreased and then increased; that is, its corrosion resistance first improved and then reduced. The lowest corrosion current density and corrosion rate of 0.3 wt % MWCNTs/WPU conductive coating among all the coatings indicated the best corrosion resistance. Its corrosion rate was about one order of magnitude lower than that of pure WPU coating. Perhaps, the MWCNTs content in WPU conductive coating prepared by electrostatic spraying was so small and the dispersibility of MWCNTs was so good that the pores arising from the film-forming process of the WPU can be filled up to a certain extent. Furthermore, due to good corrosion resistance of MWCNTs, the spatial network structure formed between MWCNTs and WPU made the coating structure denser and, thus, effectively shields the electrolyte diffusion [22].

The corrosion current density and corrosion rate of 0.6 wt % MWCNTs/WPU conductive coating were a little higher than those of pure WPU coating. Parts of the MWCNTs were likely prone to agglomeration as the MWCNT content increased and, thus, increased the number of micro-defects in the coating so that the electrolyte could diffuse easily, and could reduce the corrosion resistance of the coating.

#### 3.4.2. EIS Analysis

**Figure 4:**
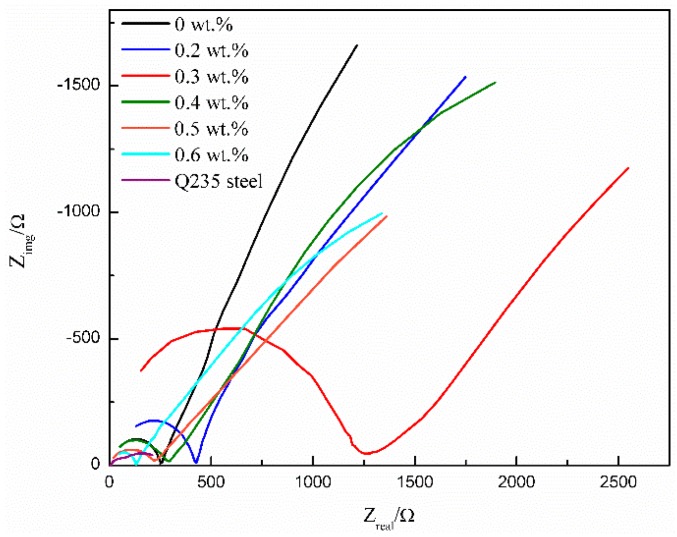
Nyquist plots of MWCNTs/WPU conductive coatings with different MWCNTs contents.

Figure 4 shows Nyquist plots of different WPU conductive coatings after immersion in 3.5 wt % NaCl solution for 17 days. A nearly complete semicircle was observed in the high frequency range on the EIS: the semicircle diameter indicated the insulation and shielding properties of the coating. The smallest semicircle diameter of the Q235 steel in the high frequency range after 17 days of immersion in 3.5 wt % NaCl solution showed that the corrosion product layer generated on the surface of Q235 steel was poorer. When the MWCNT content was 0.2–0.4 wt %, the semicircle diameter of the conductive coating in the high frequency range was greater than that of pure WPU coating. The largest semicircle diameter in the high frequency range of 0.3 wt % MWCNTs/WPU conductive coating indicated that its insulation and shielding properties were the best. The semicircle diameter of the 0.6 wt % MWCNTs/WPU conductive coating in the high frequency range was smaller than that of pure WPU coating. This shows a decrease in its shielding effect on the external environment.

**Figure 5:**
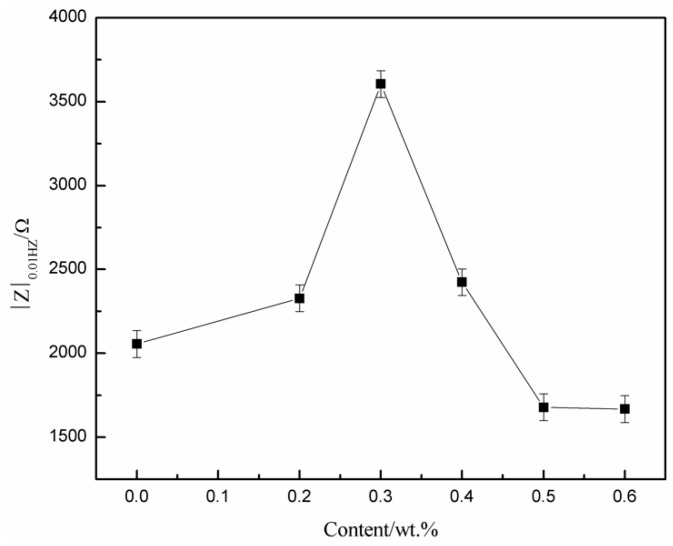
|Z|_0.01HZ_ values of MWCNTs/WPU conductive coatings with different MWCNTs contents.

A quarter semicircle in the low frequency range was in relation to the corrosion reaction between the electrolyte and the steel substrate and clearly shows that the NaCl solution had penetrated to the Q235 steel substrate. Figure 5 shows the impedance modulus |Z|_0.01HZ_ of MWCNTs/WPU conductive coating. The higher |Z|_0.01HZ_ value of the coating in the low frequency range led to the higher impedance and the stronger shielding property. As seen from Figure 5, the |Z|_0.01HZ_ value of the coating first increased and then decreased as the MWCNT content increased. When the MWCNT content was 0.2–0.4 wt %, the |Z|_0.01HZ_ values of all the conductive coatings were higher than that of pure WPU coating. The highest |Z|_0.01HZ_ value of 0.3 wt % MWCNTs/WPU conductive coating was 75.24% higher than that of pure WPU coating. A small addition of conductive filler in the polymer matrix made the corrosion resistance of the conductive coating prepared by electrostatic spraying more excellent.

### 3.5. Adhesive Strength

**Figure 6:**
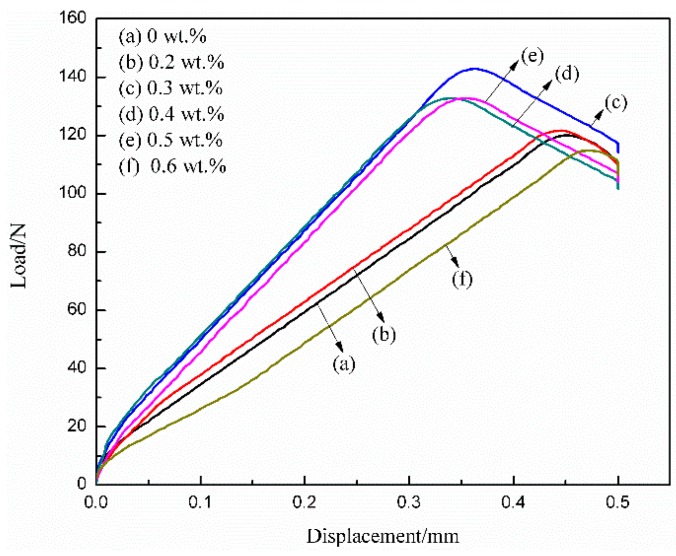
Load-displacement curves of different MWCNTs/WPU conductive coatings.

Figure 6 shows the load-displacement curves of different MWCNTs/WPU conductive coatings conducted with the results of the peel test. The maximum load at the peak of the curve was in direct proportion to the adhesive strength of the coating, which first increased and then decreased with an increase in the MWCNT content. When the MWCNT content was 0.2–0.5 wt %, the adhesive strength of the conductive coating to the Q235 steel substrate was higher than that of pure WPU coating. The largest adhesive strength of 0.3 wt % MWCNTs/WPU conductive coating (4.681 MPa) to the Q235 steel substrate was 19.99% higher than that of pure WPU coating (3.901 MPa). The reason may be that the addition of low MWCNT content was effective in preventing MWCNTs from agglomeration and sedimentation. Therefore, an increase in the contact areas of MWCNTs/WPU interfaces likely made the coating structure more compact (as seen from Figure 2a,b) and an increase in the contact areas between the WPU resin and the Q235 steel substrate improved the adhesive strength between the coating and the Q235 steel substrate [23,24]. The most compact structure of 0.3 wt % MWCNTs/WPU conductive coating just proved that a more compact structure of the coating leads to greater adhesive strength. When the MWCNT content was relatively high, parts of the agglomerated MWCNTs increased the micro-defects of the coating and, thus, generated macro-defects, and lowered the adhesive strength of the coating to Q235 steel substrates [11].

## 4. Conclusions

Different low-content MWCNTs were each dispersed in WPU by magnetic stirring and ultrasonic dispersion to prepare MWCNTs/WPU dispersions, which were each sprayed on Q235 steel substrates by an electrostatic spraying equipment to form a series of anticorrosive and conductive coatings in this work. It was concluded that a spatial network structure of MWCNTs/WPU was formed thanks to the technology of electrostatic spraying making the coating structure more compact and enhancing the combination property of the coating. The electrical conductivity, corrosion resistance, and adhesive strength of the conductive coating first increased and then decreased as the MWCNT content increased. When the MWCNT content was only 0.2 wt % (which was far lower than that of the existing conductive coatings at 1 wt %), the coating began to conduct electricity. Its resistivity was 12,675.0 Ω·m. The conductive coating with the best combination property was 0.3 wt % MWCNTs/WPU: Its resistivity was 9715.6 Ω·m, and its adhesive strength to the Q235 steel substrate was 19.99% higher than that of pure WPU coating. Its corrosion rate was only 0.0057 mm/a after an immersion in 3.5 wt % NaCl solution for 17 days. This rate was about one order of magnitude lower than that of pure WPU coating.

## Figures and Tables

**Table 1:** Resistivity of MWCNTs/WPU conductive coating with different MWCNT contents.

Sample	0.2 wt %	0.3 wt %	0.4 wt %	0.5 wt %	0.6 wt %
Square resistance (MΩ/□)	195.0 ± 3	156.2 ± 5	25.8 ± 3	4.7 ± 0.3	2.6 ± 0.2
Thickness (μm)	65.0 ± 4	62.2 ± 3	65.0 ± 4	63.5 ± 2	63.0 ± 3
Resistivity (Ω·m)	12,675.0	9715.6	1677.0	298.5	163.8

**Table 2:** The corrosion current density and corrosion rate of MWCNTs/WPU conductive coating.

Sample	0 wt %	0.2 wt %	0.3 wt %	0.4 wt %	0.5 wt %	0.6 wt %	Q235 Steel
I_0_ (A/cm^2^)	1.8706 × 10^−6^	1.7713 × 10^−6^	4.8596 × 10^−6^	7.6046 × 10^−6^	1.2015 × 10^−6^	4.3907 × 10^−6^	1.9749 × 10^−6^
Corrosion rate (mm/a)	0.022058	0.020888	0.005731	0.008968	0.014169	0.051777	2.328900
